# Serum Biomarkers in Differential Diagnosis of Idiopathic Pulmonary Fibrosis and Connective Tissue Disease-Associated Interstitial Lung Disease

**DOI:** 10.3390/jcm10143167

**Published:** 2021-07-18

**Authors:** Eva Cabrera Cesar, Lidia Lopez-Lopez, Estrella Lara, M. Victoria Hidalgo-San Juan, Concepcion Parrado Romero, Jose Luis Royo Sánchez Palencia, Elisa Martín-Montañez, Maria Garcia-Fernandez

**Affiliations:** 1 Respiratory Service, Universitary Virgen de la Victoria Hospital, 29010 Málaga, Spain; lylo_7@hotmail.com (L.L.-L.); dmhs01@hotmail.com (M.V.H.-S.J.); 2 Department of Physiology and Human Histology, Faculty of Medicine, University of Málaga, Biomedical Research Institute of Málaga, 29010 Málaga, Spain; elara@uma.es (E.L.); cparrado@uma.es (C.P.R.); igf@uma.es (M.G.-F.); 3 Department of Biochemistry, Biomedical Research Institute of Málaga, Faculty of Medicine, University of Málaga, 29010 Málaga, Spain; jlroyo@uma.es; 4 Department of Pharmacology and Paediatrics, Faculty of Medicine, University of Málaga, Biomedical Research Institute of Málaga, 29010 Málaga, Spain; emartinm@uma.es

**Keywords:** IPF, progressive pulmonary fibrosis, biomarkers

## Abstract

Introduction: The goal of this study is to determine whether Advanced glycosylated end-products (AGE), Advanced oxidation protein products (AOPP) and Matrix metalloproteinase 7 (MMP7) could be used as differential biomarkers for idiopathic pulmonary fibrosis (IPF) and connective tissue disease-associated interstitial lung disease (CTD-ILD). Method: Seventy-three patients were enrolled: 29 with IPF, 14 with CTD-ILD, and 30 healthy controls. The study included a single visit by participants. A blood sample was drawn and serum was analysed for AGE using spectrofluorimetry, AOPP by spectrophotometry, and MMP7 using sandwich-type enzyme-linked immunosorbent assay. Results: AGE, AOPP and MMP7 serum levels were significantly higher in both IPF and CTD-ILD patients versus healthy controls; and AGE was also significantly elevated in CTD-ILD compared to the IPF group. AGE plasma levels clearly distinguished CTD-ILD patients from healthy participants (AUC = 0.95; 95% IC 0.86–1), whereas in IPF patients, the distinction was moderate (AUC = 0.78; 95% IC 0.60–0.97). Conclusion: In summary, our results provide support for the potential value of serum AGE, AOPP and MMP7 concentrations as diagnostic biomarkers in IPF and CTD-ILD to differentiate between ILD patients and healthy controls. Furthermore, this study provides evidence, for the first time, for the possible use of AGE as a differential diagnostic biomarker to distinguish between IPF and CTD-ILD. The value of these biomarkers as additional tools in a multidisciplinary approach to IPF and CTD-ILD diagnosis needs to be considered and further explored. Multicentre studies are necessary to understand the role of AGE in differential diagnosis.

## 1. Introduction

Interstitial lung diseases (ILD) are a diverse group of chronic lung disorders with similar clinical and radiological manifestations and lung function tests. Idiopathic pulmonary fibrosis (IPF) and connective tissue disease-associated interstitial lung disease (CTD-ILD) are the most common forms of ILD. Although the origin of IPF is unknown, it is defined as a chronic fibrosing interstitial pneumonia with progressive functional deterioration and poor prognosis [1,2]. The diagnosis of IPF requires the exclusion of a recognisable aetiology and the identification of a radiological and/or histological pattern of usual interstitial pneumonia (UIP). Since the UIP pattern is not pathognomonic, precise IPF diagnosis can be extremely difficult. Furthermore, other chronic fibrotic lung disorders, such as CTD-ILD or chronic hypersensitivity pneumonitis, can also show a similar UIP pattern. In some cases, lung disease can occur before these systemic diseases, which also complicates the diagnosis [3,4].

Biomarkers are objectively measured, elevated indicators of physiological, pathological processes or pharmacological response to therapeutic interventions with a number of applications, including diagnosis, severity, prognosis and monitoring of treatment response. Several serum biomarkers have demonstrated potential utility for diagnosis and prognosis of ILD, but until now, use of biomarkers has not been recommended in clinical practice in IPF or CTD-ILD. Biomarkers that can help in the differential diagnosis of IPF with CTD-ILD would be very useful in identifying CTD-ILD patients with lung disease at earlier stages. An accurate diagnosis of these diseases is of key importance, considering its therapeutic and prognostic implications. Matrix metalloproteinase 7 (MMP7) is elevated in IPF compared with healthy volunteers and is one of the most promising prognostic biomarkers of this disease [5,6]. However, its ability to differentiate IPF from other ILDs is unclear [5,7].

Oxidative stress has been implicated in the pathogenesis of pulmonary fibrosis [8], although the exact mechanism remains unclear. Pulmonary redox-imbalance is suggested as playing a critical role in epithelial activation and injury of the alveolar cells, including damage to DNA, lipids and proteins, which ultimately causes severe tissue damage and fibrosis. Up to now, only a limited number of studies have assessed serum oxidative stress markers in IPF [9,10]. 

Advanced glycosylated end-products (AGE), which are formed by a combination of glycation, oxidation and/or carbonylation, are proposed as possible biomarkers [11]. They are accumulated in aging and inflammatory diseases, but also in situations of oxidative stress overload. Therefore, AGE level may be a useful biomarker of exposure to oxidative stress. AGE are involved in a number of pathologies [12,13] including pulmonary processes [14]. They cause excessive accumulation of extracellular matrix and expression of profibrotic markers such as transforming growth factor β (TGF -β) [15]. Moreover, blocking advanced AGE formation attenuates bleomycin-induced pulmonary fibrosis in rats [16].

The linking of AGE formed inside and outside cells with proteins results in local tissue damage, where the interaction of AGE with the AGE receptor (RAGE) is crucial. The implications of this interaction involve several pathological processes such as diabetes, nephropathy and rheumatoid arthritis. In the case of pulmonary consequences, AGE/RAGE is associated with chronic obstructive pulmonary disease, respiratory distress syndrome, lung cancer [17,18], and IPF [19,20].

Advanced oxidation protein products (AOPP) are elevated in several chronic inflammatory diseases with an important overload of oxidative stress [21]. In lung pathologies, high plasma levels of AOPP have been found [22,23], suggesting that serum AOPP levels could play a critical role as a biomarker for pulmonary diseases. 

Although the potential diagnostic utility of several serum biomarkers has been described for ILD, an accurate differential diagnosis of IPF and CTD-ILD has not been described. We hypothesise that AOPP, AGE and MMP7 are elevated in ILD patients compared to healthy controls. These serum biomarkers represent important factors in ILD and can be used as differential biomarkers. Even multidisciplinary experts show significant interobserver disagreement in IPF diagnosis based on high-resolution computed tomography (HRCT) and histological review, and so these results could be used to screen for ILD in a large population. Due to the importance of accurate diagnoses of ILD, and considering their therapeutic profiles and the prognostic consequences, the purpose of this study is to explore the value of serum AOPP, AGE and MMP7 levels in the differential diagnosis of IPF with CTD-ILD.

## 2. Materials and Methods

### 2.1. Patients and Control Group

Seventy-three participants, aged ≥ 18, were included in the analysis, grouped as 29 IPF, 14 CTD-ILD and 30 healthy controls. Patients were recruited from the Interstitial Lung Disease Unit at Virgen de la Victoria University Hospital (Malaga, Spain) over 56 months (Table 1). These groups were chosen because distinction between IPF and CTD-ILD is essential in order to prescribe appropriate treatment. All patients with CTD-ILD had a HRCT with a pattern of UIP. Patients with evidence of worsening or exacerbation over the previous 6 months, concomitant diagnosis of an active neoplasia, or patients who had previously received radiotherapy were excluded from the study. The healthy volunteers (Table 1) were free from signs of current infection, inflammation, or respiratory symptoms at the time of blood sampling. Informed written consent was obtained from all participants. The study was conducted according to the Declaration of Helsinki, and was approved by the Ethics in Human Research Committee of Malaga University Hospital on 26 June 2015, code 01-2015. Diagnoses were performed by multidisciplinary consensus, based on current criteria. IPF diagnosis was based on European Respiratory Society/American Thoracic Society/Japanese Respiratory Society/Latin American Thoracic Society criteria (2), whereas CTD-ILD diagnosis was based on high-resolution computed axial tomography and systemic disease criteria. [24].

### 2.2. Study Design

This was a single-centre study of patients with a multidisciplinary diagnosis of ILD. The purpose of the study was to assess whether serum markers derived from oxidative damage could serve as potential biomarkers in the differential diagnosis of ILD. We compared the serum levels of oxidative stress markers and MMP7 in IPF, and CTD-ILD patients with the levels found in healthy volunteers. The study included a single visit by participants. After providing informed consent, a blood sample was drawn from a vein (venipuncture) and a pulmonary function test was performed. Information about demographics (age, sex) and smoking habits was gathered from the clinical history. Healthy participants were paired by sex, age, and smoking status with ILD patients.

### 2.3. Sample Preparation and Marker Measurements

Whole blood was collected, after fasting for 8 h, in tubes containing EDTA at a final concentration of 50 mM. For plasma preparation, blood was centrifuged at 1200× *g* for 10 min at 4 °C and the supernatant was stored in siliconised tubes at −80 °C until use. Markers of oxidative stress were measured as follows: (i) Levels of AGE were evaluated by fluorescence spectroscopy [25]. An F-4010 spectrofluorometer (Hitachi, Tokyo, Japan) was used at 370 nm excitation and 440 nm emission wavelengths. Plasma samples were diluted with phosphate-buffered saline (PBS). Fluorescence intensity was expressed in arbitrary units (AU). (ii) AOPP were determined by spectrophotometry using a microassay adapted to Cobas Mira based on chloramine-T (ch-T) equivalents [26]. Briefly, 18 μL of sample or ch-T standard solutions (400–6.25 μmol/L) were placed in each well of the Cobas Mira AutoAnalyzer, followed by the addition of 200 μL of reaction mixture, consisting of 81% phosphate PBS, 15% acetic acid, and 4% 1.16 mM potassium iodide. Absorbance was read at 340 nm. Finally, MMP7 levels were measured by sandwich-type enzyme-linked immunosorbent assay, using a commercially available ELISA kit (MBS2021023, MyBioSource, San Diego, CA, USA) according to the manufacturer’s instruction.

### 2.4. Statistical Analysis

The variables were expressed as the mean (± SD) or n (%). To compare quantitative measures, a T-test or Mann–Whitney U-test (two group comparisons), and a one-way ANOVA or Kruskal–Wallis test (three group comparisons) were used, as appropriate. A Chi-square test was used for qualitative comparison. In order to assess the performance of AGE, AOPP and MMP7 as diagnostic markers, the area under the curve (AUC) of the receiver operating characteristic (ROC) curve was calculated. The cut-off value of serum markers was determined using the Yoden index. A significance of 5% (*p* < 0.05) was required to consider a difference to be statistically significant. Statistical analysis was performed using SPSS software version 24.0 (IBM, Chicago, IL, USA). G-power software was used for sample size calculation (Heinrich Heine University Düsseldorf, Düsseldorf, Germany). Assuming an alpha level of 0.05, a power of 80%, and 3 groups, a total of 42 participants would be necessary to provide a valid answer to the research question.

## 3. Results

### 3.1. Demographic and Clinical Characteristics

In total, 73 patients and controls were included in this study (Table 1). Of these, 29 were diagnosed with IPF, 14 with CTD-ILD in non-advanced stages, and 30 were healthy controls. A group of healthy volunteers was selected as the controls to verify whether there were differences in biomarker levels between ILD patients and healthy subjects. These three populations showed similar demographic and smoking status (Table 1), whereas forced vital capacity (FVC) in IPF and CTD-ILD patients was statistically significantly lower than in the comparison group (Table 1, *p* < 0.01). Patients with IPF received anti-fibrotic treatment, and CTD-ILD patients were treated with immunosuppressive drugs. A third of them also used oxygen therapy, distributed across both groups without any statistical differences between them. In our sample, the time from diagnosis was 38.9 ± 6.7 months. 

### 3.2. Circulating Oxidative Stress Biomarkers in Patients and Healthy Controls 

**Figure 1:**
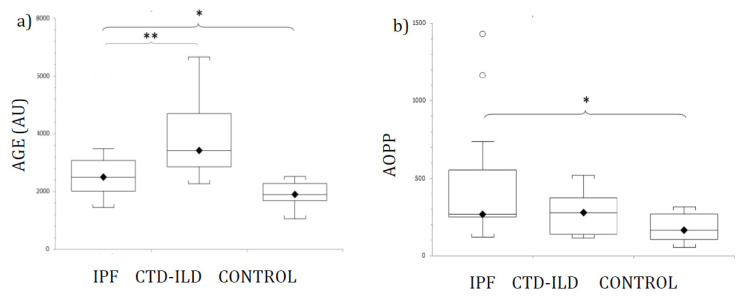
Levels of (**a**) AGE, and (**b**) AOPP in patients with IPF, CTD-ILD and healthy controls, expressed as mean ± SEM. Results were compared using one-way ANOVA with Tukey’s post hoc test. AGE: Advanced glycosylated end products. AOPP: Advanced oxidation protein products. IPF: Idiopathic pulmonary fibrosis. CTD-ILD: Connective tissue disease-interstitial lung disease. White circles represent outliers. *, **: *p* < 0.05. *: Patients versus healthy controls. **: IPF patients versus CTD-ILD patients.

Figure 1 shows AGE (Figure 1a) and AOPP (Figure 1b) plasma levels from patients and control participants. Mean levels of AGE were elevated in both ILD groups (IPF and CTD-ILD). Statistically significant differences were detected between IPF and CTD-ILD patients and healthy controls. Furthermore, AGE was also significantly elevated in CTD-ILD compared with the IPF group (*p* < 0.03). Mean plasma AGE concentrations and *p*-values for IPF and CTD-ILD groups in comparison with the control group (mean AGE plasma concentration 1918.5 ± 145.7 AU) were as follows: IPF, 2728.9 ± 300.4 AU (*p* < 0.02); CTD-ILD, 3917.1 ± 566.3 AU (*p* < 0.002).

AOPP plasma levels were significantly higher in both ILD groups compared with healthy controls. However, no significant difference in AOPP levels was detected between IPF and CTD-ILD patients. Actual mean plasma AOPP concentrations and *p*-values for ILD groups in comparison with the control group (mean AOPP plasma concentration 173.8 ± 24.7 µM) were as follows: IPF 469.7 ± 121.1 µM (*p* < 0.02); CTD-ILD 275 ± 50.1 µM (*p* < 0.02).

### 3.3. AGE and AOPP as Diagnostic Markers for IPF and CTD-ILD

ROC analysis was performed and the best cut-off values chosen for AGE and AOPP were 2370 AU and 202.5 µM, respectively. For AGE, AUC values were 0.78 (95% CI 0.60–0.97 *p* < 0.001) for IPF (Table 2), and 0.95 (95% CI 0.86–1) for CTD-ILD patients (Table 3). The sensitivities and specificities when using these cut-off values are shown in Table 2 and Table 3.

### 3.4. Circulating MMP7 in Patients and Healthy Controls 

**Figure 2:**
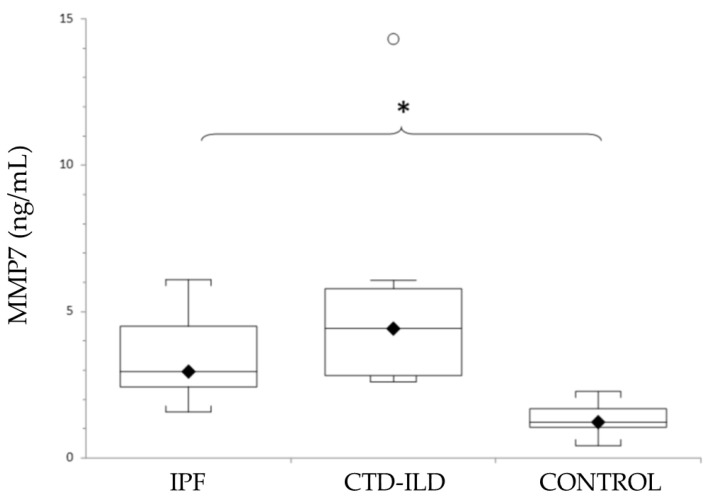
Levels of MMP7 in patients with IPF, CTD-ILD and healthy controls, expressed as mean ± SEM. Results were compared using one-way ANOVA with Tukey’s post hoc test. MMP7: Matrix metalloproteinase 7. IPF: Idiopathic pulmonary fibrosis. CTD-ILD: Connective tissue disease-interstitial lung disease. White circles represent outliers. *: *p* < 0.05. *: Patients versus healthy controls.

As shown in Figure 2, mean levels of MMP7 were elevated in both ILD groups (IPF and CTD-ILD). Thus, a statistically significant difference was detected between both IPF patients and CTD-ILD patients and the control group. Although the MMP7 levels were higher in patients with CTD-ILD compared with IPF, no statistically significant difference was detected. Actual mean plasma MMP7 concentrations and *p*-values for IPF and CTD-ILD groups in comparison with the control group (mean MMP7 plasma concentration 1.4 ± 0.15 ng/mL) were as follows: IPF, 3.13 ± 0.38 ng/mL (*p* < 0.0001); CTD-ILD, 5.14 ± 1.23 ng/mL (*p* < 0.0001).

### 3.5. MMP7 as Diagnostic Markers for IPF and CTD-ILD

ROC analysis reveals an AUC of 0.96 (95% IC 0.91–1, *p* < 0.001) for IPF and AUC of 1 (95 IC 1–1, *p* < 0.001) for CTD-ILD (Table 2). The best cut-off value was 2.07. For IPF patients, the sensitivity was 92.3% and the specificity was 92.9%. In the case of CTD-ILD patients, sensitivity was 100% and specificity was 92.9% (Table 2 and Table 3). The combined biomarker analysis of AGE and MMP7 shows a sensitivity of 93.33% and a specificity of 100%, when differentiating between IPF and CTD-ILD. It also shows a positive predictive value of 100 and negative predictive value of 96.8 (95% IC 81.3–99.6).

## 4. Discussion

We assessed the potential values of AGE, AOPP and MMP7 as diagnostic biomarkers for IPF and CTD-ILD by comparing serum concentrations in IPF and CTD-ILD patients with healthy controls, and by comparing CTD-ILD and IPF patients. Our data show serum levels of all markers in IPF or CTD-ILD patients that are significantly higher than those in healthy participants. Moreover, AGE was also significantly elevated in CTD-ILD compared with the IPF group. Although no significant differences in MMP7 and AOPP levels were detected between CTD-ILD and IPF patients, the levels of MMP7 were more elevated in CTD-ILD patients than in the IPF group, and, conversely, AOPP levels were more elevated in IPF patients than in the CTD-ILD group. Using MMP7 to diagnose IPF or CTD-ILD resulted in the highest sensitivities and specificities. 

Differential diagnosis for ILDs is complex due to their similar morphological, clinical, and radiological characteristics. Since the appearance of specific anti-fibrotic treatments for IPF [27,28], accurate differential diagnoses at earlier stages are especially important, and the identification of serum biomarkers can help. It is of special interest in those CTD-ILD patients with a HRCT pattern of UIP, which is the case of our patients, and in those without a finding of autoimmunity that indicates specific CTD pathology.

We therefore explore biomarkers that can support the differential diagnosis of IPF with CTD-ILD, which is of key importance considering its therapeutic implications. Thus, combined immunosuppressive treatment shows negative effects in IPF patients [29], and lung complications can appear at earlier stages of CTD-ILD before systemic disease, complicating the therapeutic approach [24]. Even though the use of anti-fibrotics in progressive ILD has been proposed, an accurate diagnosis is still required [30].

To date, several lung circulating biomarkers have been studied for ILD diagnosis, staging of disease, and prognosis. Although a large number of these molecules have shown utility, the routine use of such biomarkers is not recommended in diagnostic guidelines or routinely used in clinical practice. For example, Krebs von den Lungen (KL-6), surfactant proteins (SP-A and SP-D), MMPs (MMP1 and MMP7), extracellular collagen fragments generated by MMPs and released into circulation (neoepitopes), C-C motif chemokine ligand 18 (CCL18), C-X-C motif chemokine 13 (CXCL13), heat shock protein 47 (HSP47), insulin-like growth factor binding proteins (IGFBP1 and 2), and periostin [31,32,33,34]. Furthermore, telomere shortening has been associated with poor prognosis and increased risk of death [35,36].

In this study, significantly higher plasma AOPP levels in ILD patients were observed as compared to healthy controls. ROC analysis showed that AOPP serum levels moderately distinguished either IPF or CTD-ILD patients from healthy controls. Regarding the levels of AOPP in patients with lung fibrosis, Servetazz et al. measured AOPP serum levels in patients with limited or diffuse Systemic sclerosis (SSc). They found significantly increased AOPP levels in patients with diffuse SSc and lung fibrosis, whereas serum AOPP concentrations in patients with limited cutaneous SSc and no lung fibrosis did not differ from concentrations observed in healthy controls [37]. As in our study, bleomycin administration, to induce pulmonary fibrosis in animals, caused significant increases in AOPP compared to the sham group [38]. Although we have explored AOPP levels as a potential biomarker in the differential diagnosis of ILD, we have not found significant differences between AOPP levels in IPF and CTD-ILD patients.

Similarly, the oxidative stress marker AGE was significantly more abundant both in IPF and CTD-ILD patients compared with healthy participants. Our data show AGE serum levels in CTD-ILD patients are significantly higher than those found in IPF patients (43.6% higher). The AGE levels in IPF patients were also significantly more elevated when compared with the healthy group. Elevated AGE levels in CTD-ILD patients, compared to IPF patients, may be due to collagen alterations related to CTD, where AGE directly affects collagen properties [39,40]. The AGE/RAGE axis is thought to mediate inflammation in several autoimmune processes [41], making it a probable therapeutic target for these diseases [42]. These data suggest that serum AGE levels could not only differentiate between ILD and healthy people, but also between IPF and CTD-ILD patients.

Previous studies showed significantly increased serum AGE or soluble RAGE (sRAGE) levels and decreased alveolar epithelial cell RAGE expression in IPF patients compared with healthy participants [14] [19,20]. Concerning the study of AGE/RAGE in other ILDs, Manichaikul et al. [43] found significantly lower plasma sRAGE levels in patients with IPF and other ILDs (including CTD-ILD) when compared with healthy controls. Our study suggests the possible use of AGE as a differential diagnostic biomarker that could support diagnosis of IPF and CTD-ILD and their proper therapeutic profiles.

MMP7 and its utility for diagnosis and prognosis is one of the best studied serum biomarkers in IPF and SSc-ILD. Although it is categorised by its major mechanistic pathways, extracellular matrix remodelling, a link between oxidative stress and MMP has been identified [44]. In this study, significantly higher plasma MMP7 levels in ILD patients were observed when compared with healthy controls. ROC analysis showed that MMP7 serum levels clearly distinguished both IPF and CTD-ILD patients from healthy controls in our study, thus making them a promising biomarker to differentiate between ILD patients and healthy controls. Until now, several studies have demonstrated that MMP7 is a valuable biomarker for IPF alone, or in combination with other candidate biomarkers, such as MMP1, MPP8, IGFBP1, TNF SP-A, SP-D or KL-6 [5,6,45,46], but not for CTD-ILD.

The small sample size for the clinical entities considered in our analysis and the cross-sectional database are the mains limitations. This does not allow the effect of tobacco on the levels of these biomarkers to be studied. Our work is a single-centre study, which limits its external validity. The value of these biomarkers as additional tools in a multidisciplinary approach to IPF and CTD-ILD diagnosis needs to be considered and further explored. Undoubtedly, multi-centre and prospective studies are necessary to understand the role of AGE in IPF and CTD-ILD differential diagnosis, as well as its possible relationship to imaging and respiratory function tests.

## 5. Conclusions

In conclusion, our results provide support for the potential value of serum AGE, AOPP, and MMP7 concentrations as diagnostic biomarkers in IPF and CTD-ILD, with MMP7 being one of the most valuable biomarkers for differentiating between ILD patients and healthy controls. The differential diagnosis between ILDs requires important clinical implications, and this study demonstrates, for the first time, the possible use of AGE as a differential diagnostic biomarker between IPF and CTD-ILD. Due to the similar morphological, clinical, and radiological characteristics of IPF and CTD-ILD, accurate differential diagnoses at earlier stages are especially important, given the therapeutic and prognostic implications.

## Figures and Tables

**Table 1:** Patient demographics and clinical characteristics. Continuous variables are expressed as the mean (± SD). Categorical data are expressed as *n* (%). IPF: Idiopathic pulmonary fibrosis. CTD-ILD: Connective tissue disease-associated interstitial lung disease. FVC: Forced vital capacity. DLCO: Diffusing capacity of the lung for carbon monoxide. NS: non-significant. NE: not evaluated. *: IPF versus healthy controls. **: CTD-ILD versus healthy controls.

Characteristics	IPF	CTD-ILD	Controls	*p* Value
Patients n	29	14	30	
Age, years	63.3 ± 2.7	60 ± 2.9	58.3 ± 3.5	NS
Male%	19 (65.5)	6 (42.9)	18 (60)	NS
Smoking status				
Active or former smokers	21 (72.4)	11 (78.6)	20 (66.7)	NS
Pulmonary function				
FVC, %predicted	62.6 ± 15 *	62 ± 18.8 **	101.8 ± 20	<0.01
DLCO, %predicted	44.4 ± 15.2	47.5 ± 12.2	NE	

**Table 2:** ROC curves analysis to distinguish IPF from healthy participants. AGE: Advanced glycosylated end products. AOPP: Advanced oxidation protein products. MMP7: Matrix metalloproteinase 7. AUC: Area under the curve.

Parameter	Sensitivity (%)	Specificity (%)	AUC	95% CI	*p* Value
AGE (AU)	71.4	80	0.78	0.60–0.97	<0.001
AOPP (µM)	83.3	69.2	0.80	0.63–0.98	<0.001
MMP7(ng/mL)	92.3	92.9	0.96	0.91–1	<0.001

**Table 3:** ROC curves analysis to distinguish CTD-ILD from healthy participants. AGE: Advanced glycosylated end products. AOPP: Advanced oxidation protein products. MMP7: Matrix metalloproteinase 7. AUC: Area under the curve.

Parameter	Sensitivity (%)	Specificity (%)	AUC	95% CI	*p* Value
AGE (AU)	85.7	80	0.95	0.86–1	<0.001
AOPP (µM)	55.5	69.2	0.71	0.63–0.98	<0.001
MMP7(ng/mL)	100	92.9	1	1–1	<0.001

## Data Availability

The datasets during and/or analysed during the current study are available from the corresponding author on reasonable request.
